# Identification of immune subtypes of Ph-neg B-ALL with ferroptosis related genes and the potential implementation of Sorafenib

**DOI:** 10.1186/s12885-021-09076-w

**Published:** 2021-12-14

**Authors:** Yang Hong, Ling Zhang, Xiaopeng Tian, Xin Xiang, Yan Yu, Zhao Zeng, Yaqing Cao, Suning Chen, Aining Sun

**Affiliations:** 1 Department of Hematology, The First Affiliated Hospital of Soochow University, Jiangsu Institute of Hematology, National Clinical Research Center for Hematologic Diseases, Suzhou, China; 2 Institute of Blood and Marrow Transplantation, Collaborative Innovation Center of Hematology, Soochow University, Suzhou, China

**Keywords:** Ferroptosis, Acute lymphoblastic leukemia, Unsupervised clustering, Sorafenib, Immune

## Abstract

**Background:**

The clinical outcome of Philadelphia chromosome-negative B cell acute lymphoblastic leukemia (Ph-neg B-ALL) varies considerably from one person to another after clinical treatment due to lack of targeted therapies and leukemia’s heterogeneity. Ferroptosis is a recently discovered programmed cell death strongly correlated with cancers. Nevertheless, few related studies have reported its significance in acute lymphoblastic leukemia.

**Methods:**

Herein, we collected clinical data of 80 Ph-neg B-ALL patients diagnosed in our center and performed RNA-seq with their initial bone marrow fluid samples. Throughout unsupervised machine learning K-means clustering with 24 ferroptosis related genes (FRGs), the clustered patients were parted into three variant risk groups and were performed with bioinformatics analysis.

**Results:**

As a result, we discovered significant heterogeneity of both immune microenvironment and genomic variance. Furthermore, the immune check point inhibitors response and potential implementation of Sorafenib in Ph-neg B-ALL was also analyzed in our cohort. Lastly, one prognostic model based on 8 FRGs was developed to evaluate the risk of Ph-neg B-ALL patients.

**Conclusion:**

Jointly, our study proved the crucial role of ferroptosis in Ph-neg B-ALL and Sorafenib is likely to improve the survival of high-risk Ph-neg B-ALL patients.

**Supplementary Information:**

The online version contains supplementary material available at 10.1186/s12885-021-09076-w.

## Background

B cell acute lymphoblastic leukemia (B-ALL) diagnosis and treatment had achieved remarkable improvement over the past decades. Thanks to the discovery of tyrosine kinase inhibitor (TKI), the survival of Philadelphia chromosome-positive B cell acute lymphoblastic leukemia was significantly prolonged [1]. However, more than 50% of B-ALL patients were negative in Philadelphia chromosome screening [2] and the prognosis of Philadelphia chromosome-negative B cell acute lymphoblastic leukemia (Ph-neg B-ALL) is heterogeneous [3]. Although chimeric antigen receptor T cells (CAR-T) therapy specifically targeting B cell antigens such as CD19 and CD22 benefited (for some cases) of refractory or relapsed B-ALL (R/R B-ALL), exhaustion and relapse of CAR-T after CAR-T therapy had limited its long-term efficiency [4]. Consequently, the exploration of new mechanisms involving Ph-neg B-ALL and therapeutic targets are crucially needed.

Ferroptosis was identified back in 2012 by Dixon [5], it is a form of cell death characterized by an overwhelming, iron-dependent accumulation of lethal lipids and reactive oxygen species (ROS) [6]. Several studies have confirmed that ferroptosis leads to tumor cells death and inhibits tumor growth [7–9]. On the other hand, apoptosis as another well-known form of cell death has been extensively investigated in the past 30 years while the clinical implementation of drugs targeting apoptosis regulators in cancers still faces some challenges [10]. Therefore, targeting recently identified ferroptosis processes might provide an efficient way to suppress tumor growth especially in tumors resistant to apoptosis inducers.

Although, understanding ferroptosis entirely is far from completely clear, researchers have identified several genes strongly correlated to ferroptosis progress. However, the core role of ferroptosis in Ph-neg B-ALL remained unclear. In this study, we aimed to explore the potential involvement of ferroptosis in the Ph-neg ALL patients with 24 ferroptosis related genes (FRGs) reported in the former research [11]. Primarily, we planned to evaluate prognostic significance of the FRGs in the Ph-neg ALL patients with unsupervised clustering and perform the bioinformatics-based analysis to reveal the mechanism of ferroptosis-involved genetic and biological heterogeneity. Secondly, whether the variant degrees of ferroptosis involvement correlated with immune microenvironment of leukemia was the other theme of our research for the emerging role of immune therapies in the field of cancers. Lastly, we expected to find out a certain kind of ferroptosis-inducers to treat high-risk Ph-neg ALL patients potentially.

## Methods

### Patients

A total of 80 patients diagnosed as de novo Ph-neg B-ALL were admitted in our center between October 2015 and January 2021. The Philadelphia chromosome identification was verified by both chromosome R-banding technique and fluorescence in situ hybridization (FISH). Additionally, we collected the enrolled patients’ initial bone marrow fluid samples from the clinical biological sample database of our center. Our study was approved by the ethics board of the First Affiliated Hospital of Soochow University and performed in agreement with the Declaration of Helsinki. All patients signed consent forms and the median follow-up time was 23.5 months.

### Targeted gene mutational analysis

Genomic DNA was extracted from BM (Invitrogen) at the diagnosis phase and further processed as described in our previous report [12]. Summarily, targeted genomic sequencing of 172 leukemia recurrent mutated genes (listed in Table S2) was performed using the Ion S5 system (Personal Genome Machine, ThermoFisher, Grand Island, NY, USA) in 80 Ph-neg B-ALL patients and the trusted gene mutations were annotated after the filtration of synonymous and located variants outside coding sequence (CDS).

### Whole transcription sequencing (RNA-seq) and data processing

To explore the potential mechanism related to the prognosis of Ph-neg B-ALL, we extracted total RNA with Trizol reagent and ensured the qualification of each RNA sample. Furthermore, total transcriptome RNA sequencing (RNA-seq) was performed with qualified extracted RNA samples. Concisely, we first established the library of each sample according to the protocol recommended by NEBNext® Ultra™ RNA library Prep Kit for Illumina®. Subsequently, we quantified the libraries by both Qubit 3.0 and Agilent 2100, and then ensured the effective concentration of each library more than 10 nM through fluorescent quantitative PCR (qPCR). Lastly, these libraries were sequenced on the HiSeq sequencing platform after clustering by Hiseq PE Cluster Kit v4-cBot-HS.

To generate the gene expression data for the upcoming analysis, we filtered the raw sequencing data to remove joint sequences and bad-qualified results in the first step. Then the filtered data were annotated in the HISAT2 software with the reference file downloaded from ENSEMBL database (http://www.ensembl.org/index.html). Finally, reads count for each gene in the above samples was counted by HTSeq v0.6.0 and fragments per kilobase million mapped reads (FPKM) was then calculated to represent the expression level of genes in each sample. The formula is shown as: $\mathrm{FPKM}=\frac{10^6\ast F}{NL/{10}^2}$. (F is the number of fragments in a certain sample that is assigned a certain gene, N is the total number of mapped reads in the certain sample and L is the length of the certain gene.)

### Oncomine analysis

In the purpose of evaluate the ferroptosis role in B-ALL, we visited the Oncomine database (https://www.oncomine.org/resource) and performed the FRGs’RNA-level meta-analysis to the comparisons between the B-ALL samples and the normal controls in multiple B-ALL datasets [13–15]. The significance of FRGs variance was computed in the form of -log10 (***P***-value).

### K-means clustering

K-means clustering is one of the most popular algorithms of unsupervised machine learning, processed with the Scikit-learn package V0.24.2 in Python V3.8, the FPKM values of 24 FRGs in 80 Ph-neg B-ALL samples were standardized in the range of [0,1] before clustering to eliminate the influence of dimension and variation range. Thereafter, the dimension was reduced from 24 to 2 after the principal component analysis (PCA). Furthermore, the most significant K value was determined with the ‘elbow’ method. Eventually, a total of 80 samples were parted into variant groups according to the K-means clustering results. To assess the clustering models, we utilized the adjusted rand index (ARI), the adjusted mutual index (AMI), the V-measure score, the Fowlkes–Mallows index (FMI), the Silhouette Coefficient and the Calinski-Harabaz index.

### Immune characteristics of the sample clusters

To investigate the variance of the immune infiltration in between the clusters, the RNA-seq data were processed with the CIBERSORTx algorithm [16]. A number 22 variant immune cells infiltration levels were calculated, and the results adequate with ***P***-value < 0.05 were then adopted for further analysis. K means clustering was also used in the abovementioned way to divide 80 qualified samples into five components according to the immune infiltration results to demonstrate the feature differences among the three groups with variant degrees of risk.. Five was chosen as the k-value to perform the clustering to ignore the immune cells with extremely low infiltration and to appear the making-up difference of the samples with diverse immune infiltration in the variant groups apparently. We further utilized the ESTIMATE algorithm to estimate the purity of the tumor, and the R package “estimate” for storm and the immune cells ratio, we then utilized ESTIMATE score to evaluate immune statement of leukemia’s micro-environment [17]. The cytolytic score generated from the average log10 value of five granzymes and perforin-1 (PRF1) gene expressions and the inflammatory score calculated in former reports [18, 19] were assembled to reveal the cytotoxic immune cell activity. The response to PD-1 blockage therapy was estimated according to a previous reported formula [20].

### Gene enrichment analysis

Different expression genes (DEGs) amongst the sample clusters were calculated with R package “edgeR”. The Gene oncology and KEGG pathway enrichment were annotated with R packages “clusterProfiler” and “enrichplot” based on FDR < 0.05 and |logFC| > 2 DEGs. The adjusted enriched terms at a ***P*** value< 0.05 were accepted. The Immune enrichment was calculated with package “ClueGO” using the Cytoscape software [21]. To explore the different involvement degree of ferroptosis, three modules of ferroptosis regulated genes (drivers, suppressors and markers) were downloaded from the FerrDb database [22] to merge with FDR < 0.05 and |logFC| > 1 DEGs.

### Sorafenib sensitivity evaluation

The validated Sorafenib related genes established by former researches were downloaded from the Comparative Toxicogenomics Database (CTD) [23]. Six genes (FLT3 positively while the 5 genes left negatively correlated to the susceptibility to Sorafenib) were picked and the sum of the six genes log10 (FPKM) was defined as Sorafenib sensitivity score to evaluate the susceptibility to Sorafenib among variant sample clusters. To validate the efficiency of this score system, we utilized both gene expression data of 1457 common cell lines provided from the Broad Institute Cancer Cell Line Encyclopedia (CCLE) (https://portals.broadinstitute.org/ccle) and half maximal inhibitory concentration (IC50) data of Sorafenib in multiple cell lines from the Genomics of Drug Sensitivity in Cancer (GDSC) [24].

### Development and validation of a prognostic model based on 8 FRGs

To utilize the prognosis value of FRGs, we first employed a Cox regression analysis to calculate both hazard ratios (HRs) and ***P*** values. FRGs that has a ***P*** value < 0.1 was adopted for further analysis. Thereafter, the least absolute shrinkage and the selection operator (LASSO) Cox regression model were employed to narrow down the candidate genes and to develop the survival prediction model. As a final step, a total 8 FRGs along with their coefficients were retained, and the penalty parameter (λ) was limited by the minimum criteria. 70% of the samples were randomly selected to build the prediction model, while the 30% left was utilized as the validation cohort. The areas under curves (AUCs) of 1 year and 3 years survival were calculated with the R package ‘time ROC’. The Calibration curve was drawn to assess the efficiency of prediction. Subsequently, both the development and the validation cohorts survival were estimated by the Kaplan-Meier method and the difference significance in survival between high and low risk groups was evaluated by means of a stratified log-rank test.

### Statistical analysis

All statistical analyses were completed using R software (v4.0.2), Graph Pad Prism 8.0 (GraphPad Inc., San Diego, CA, USA) and Python software (v3.8). We then administered a two-way Student T test to perform a numerical comparison and a Chi-square test to analyze the categorical data to compare the clinical and the molecular parameters between groups. Univariate Cox regression was conducted to determine factors with an independent prognostic value. Multivariate Cox regression model was established as mentioned above. ***P*** value of < 0.05 was considered to be statistically significant and was presented as *P < 0.05, **P < 0.01, or ***P < 0.001.

## Results

### Expression characteristics of FRGs in ALL

**Fig. 1:**
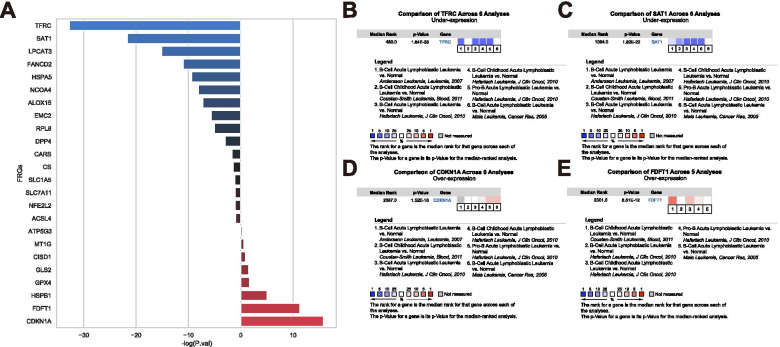
The meta-analysis of 24 FRGs expression in B-ALL compared to normal people in Oncomine database. **A** The -log10 of the ***P*** values after meta-analyses were shown. The results of over-expression genes remained the raw values while the results of rest under-expression genes were taken negative values of the raw data. The bars were shaded with variant colors to represent the expression difference of FRGs (Red: over-expression; Blue: under-expression; Black: no significant difference). **B**, **C** The top down-expressed genes TFRC and SAT1 results were shown. **D**, **E** The top over-expressed genes CDKN1A and FDFT1 results were captured

After performing the meta-analysis of the FRG-expressing difference between the B-ALL datasets and the datasets of normal controls in the Oncomine database, significant difference was noticed in 17 out of 24 FRGs (***P*** < 0.05) indicating that the ferroptosis mechanism might play a potential role in the B-ALL development (Fig. 1A). In a more thorough way, the activity of the transferrin receptor (TFRC) and the permidine/spermine N1-acetyltransferase1 (SAT1) where the functions related the proferroptosis were significantly inhibited (Fig. 1B, C). Meanwhile, the cyclin-dependent kinase inhibitor1 (CDKN1A) and the farnesyl-diphosphatefarnesyltransferase 1 (FDFT1) expression were significantly enhanced which might promote an antiferroptotic effect (Fig. 1D, E).

### Variant risk clusters based on 24-FRGs expression

**Fig. 2:**
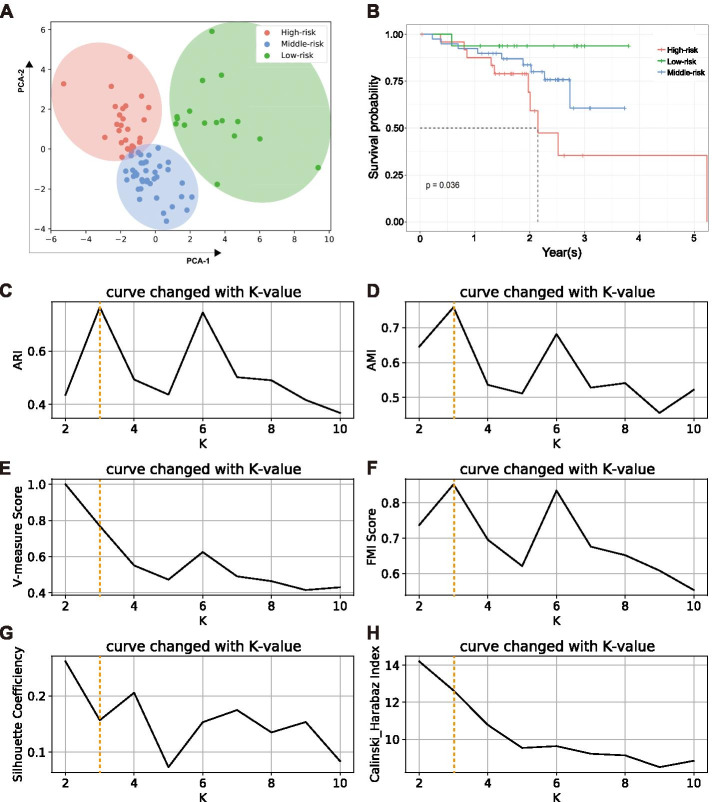
80 Ph-neg B-ALL patients were clustered into three groups by K-means clustering based on FRGs. **A** 80 dots representing enrolled Ph-neg B-ALL patients after PCA dimension reduction were located on two-dimensioned plane and circled into three groups according to the labels of the Kmeans clustering result. **B** The Kaplan-Meier analysis performed to three clusters was shown and there was significant difference among three clusters (***P*** = 0.036). **C**-**H** Six common evaluation methods were adopted to demonstrate K = 3 was an ideal K value

Powered by K-means clustering, 80 Ph-neg B-ALL samples were divided into three variant risk clusters (Fig. 2A). Combined with our clinical follow-up data, three clusters had significant survival difference (***P*** = 0.036) (Fig. 2B). The model evaluation through k varying from 2 to 10, k = 3 was noted as best k value in 3 out of 6 methods while the remaining methods indicated that k = 2 was acceptable as well (Fig. 2C-H). Consequently, 25 samples were clustered as the ‘High-risk’ group and 39 samples were included into the ‘Middle-risk’ group. The remaining 16 samples were defined as the ‘Low-risk’ group according to overall survival status.

### Clinical and genetic characteristics of Ph-neg B-ALL patients

The included samples were clustered into a ‘High-risk’ group (*n* = 25), a ‘Middle-risk’ group (*n* = 39) and a ‘Low-risk’ group (*n* = 16) based on the K-means clustering results. The median age in all patients was 26 years old (range 9–56). After performing a systematic analysis of the clinical data, three groups had significant differences in their molecular alterations (***P*** = 0.0269) while no apparent differences in the complete blood count (CBC), the bone marrow blasts ratio, the immunophenotyping and the chromosome karyotypes was noticed. Meanwhile, the treatment including CAR-T therapy and hematopoietic stem cell transplantation (HSCT) also had no major difference. Information in details was provided in Table S1.

### Gene correlated to ferroptosis tended to mutate in the ‘high-risk’ group

In order to decipher the genomic varietal spectrum of Ph-neg B-ALLs, all patients in our study underwent Next Generation Sequencing (NGS) with a panel of 172 recurrent gene targets in hematologic malignancies. These variants were detected in 66 out of the 80 patients (82.5%) and the median number of variants per patient was 2 (range, 0–11). A total of 15 out of these 80 (18.8%) patients carried one, and 51 (28.8%) patients harbored two (*n* = 23), three (*n* = 7) or at least four (*n* = 21) variants. The most frequently mutated genes were NRAS (*n* = 20, 25.0%), KRAS (*n* = 12, 15.0%), SETD2 (*n* = 10, 12.5%), FLT3 (*n* = 8, 10.0%), PTEN11 (*n* = 8, 10.0%), and TP53 (*n* = 8, 10.0%).

**Fig. 3:**
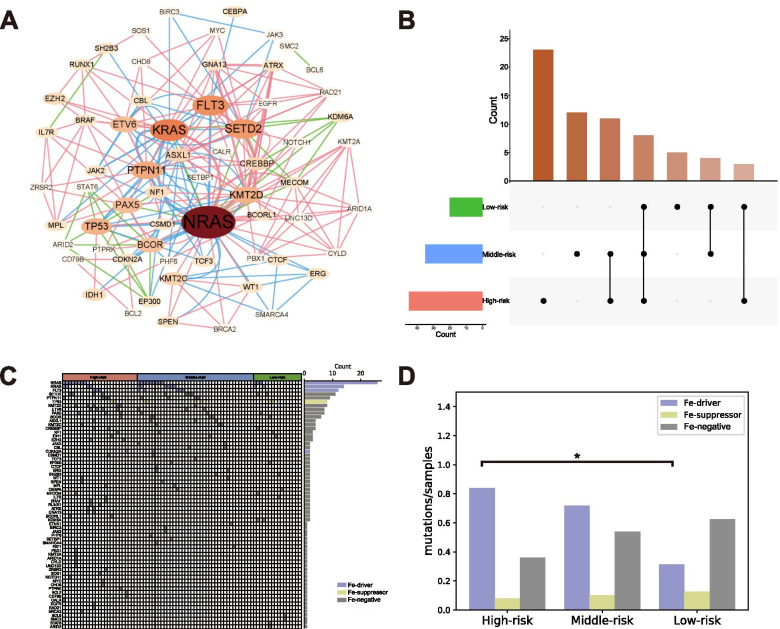
Genetic characteristics of 80 Ph-neg B-ALL patients and ferroptosis involvement analysis. **A** Coordinated mutation network. Their coordinated mutation relationships in three clusters (red: High-risk, blue: Middle-risk, green: Low-risk) and the mutated frequency of each gene (in the form of both node size and dark degree) were revealed. **B** The heterogeneity of mutations in three clusters was shown in the set-up picture after statistics. **C** The spectrum of gene mutations in 80 Ph-neg B-ALL patients clustered into three groups. Referred to FerrDb, NRAS, KRAS, FLT3 and CDKN2A were annotated as ferroptosis driver genes (Fe-driver) while TP53 was annotated as a ferroptosis suppressor gene (Fe-suppressor). The rest mutated genes lacked of evidence correlated to ferroptosis were annotated as the genes negatively related to ferroptosis (Fe-negative). **D** To compare the ferroptosis related gene mutation among three groups, the accumulated mutation counts were divided by sample counts in variant groups separately. The number of Fe-drivers was significantly different between ‘High-risk’ group and ‘Low-risk’ group (***P*** = 0.043)

Parted in three clusters, the ‘High-risk’ group contained 45 variant mutated genes; there were 35 and 20 different mutated genes in the ‘Middle-risk’ and the ‘Low-risk’ groups separately. Considering the coordination of gene mutations classified in these groups, there was also apparent heterogeneity. We defined the connection between two mutations in the same sample as one ‘edge’. As a result, the ‘High-risk’ group occupied 147 edges, while the ‘Middle-risk’ group and the ‘Low-risk’ groups included 67 and 26 edges separately (Fig. 3A). Considering the differences in the group size, we standardized the results into 5.88 (High-risk), 1.72 (Middle-risk), and 1.63 (Low-risk) edges per mutation and demonstrated that more coordinated mutations existed in the ‘High-risk’ group than the other groups. After the horizon comparison of mutations in three groups, the classifications of mutations in the descending order were the single ‘High-risk’ group, the single ‘Middle-risk’ group, the ‘High-risk’ & ‘Middle-risk’ group, the ‘High-risk’ & ‘Middle-risk’ & ‘Low-risk’ group, the single ‘Low-risk’ group, the ‘Middle-risk’ & ‘Low-risk’ group and the ‘High-risk’ & ‘Low-risk’ group (Fig. 3B).

Due to the machine-learning clustering based on FRGs, the interfered genes correlated to ferroptosis were likely to be varying in the genomics among different groups. In order to validate our hypothesis, we acquired ferroptosis-correlated genes from the FerrDb. According to the Ferrdb classification, the genes were annotated as drivers, suppressors and markers. In details, the ferroptosis drivers are genes that promote ferroptosis. The ferroptosis suppressors are genes that prevent ferroptosis and the ferroptosis markers are genes that indicate the occurrence of ferroptosis. After merging our mutation data with the genes from the FerrDb, NRAS, KRAS, FLT3 and the CDKN2A, they were defined as the ferroptosis drivers (Fe-driver) while TP53 was defined as the ferroptosis suppressor (Fe-suppressor). The remaining mutation genes were named as Fe-negative genes since there was no sufficient evidence to connect them with ferroptosis. Upon gathering the mutation data, we surprisingly found that Fe-drivers and Fe-suppressors were the high recurrent gene mutations in our gene mutation detection panel (Fig. 3C). Further analysis indicated that the Fe-diver gene mutations existed in the ‘High-risk’ group were much more significant compared to those in the ‘Low-risk’ group after the scandalization with the sample counts (***P*** = 0.043) (Fig. 3D).

### Infiltrated immune cells difference among subgroups

**Fig. 4:**
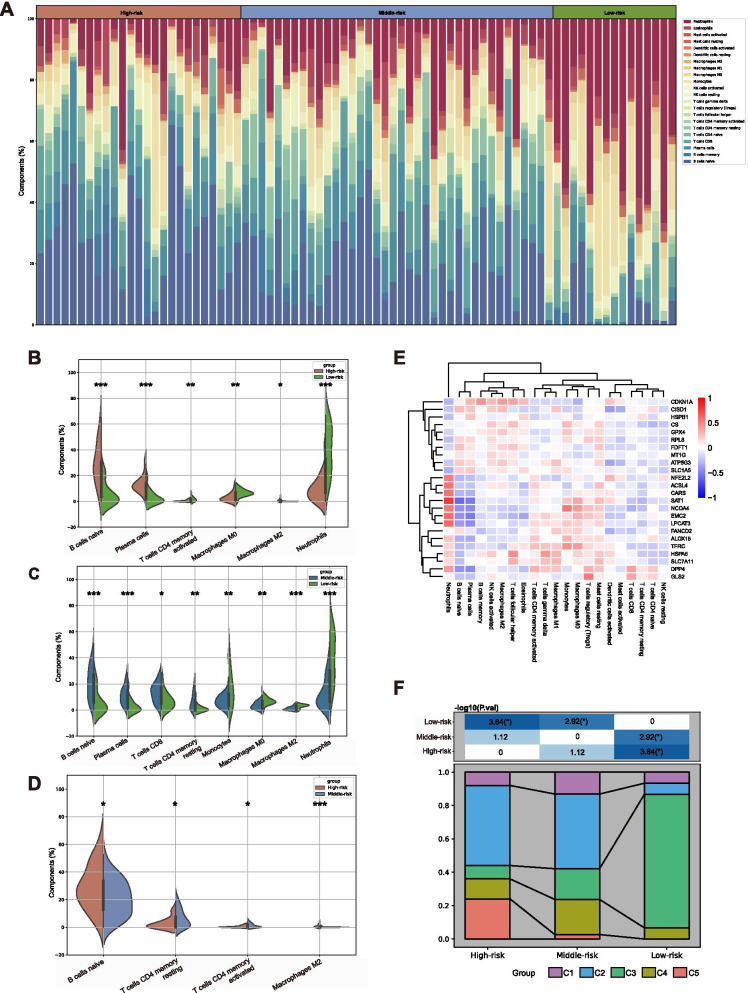
The Immune infiltration variance among the three clusters and their correlation with ferroptosis. **A** The infiltration statements of 22 variant types of immune cells of each sample were calculated with CIBERSORTx algorithm. **B**-**D** The significant differences of immune cells infiltration between any two groups from three variant risk clusters were shown in the violin pictures. **E** The relationship between the immune infiltration and FRGs was calculated with Pearson correlation coefficient. **F** After clustering 22 infiltrated immune cells in 5 clusters (C1 to C5), we found both the difference (−log10 (***P***.val) =3.84) between ‘High-risk’ group and ‘Low-risk’ group and the difference (−log10 (***P***.val) =1.12) between ‘Middle-risk’ group and ‘Low-risk’ group were significant

Subsequently, we further explored the immune cell infiltration statement amongst the subgroups. Equipped with the CIBERSORTx method, 22 common immune cells were analyzed in 80 variant samples and excluded two samples where ***P*** value failed to meet the acquisition of ‘< 0.05’ (Fig. 4A). In details, the B cells naïve plasma cells, the T cells CD4 memory activated, the macrophage M0, the macrophage M2 and the neutrophils were significantly dissimilar between the ‘High-risk’ and the ‘Low-risk’ groups (Fig. 4B). Meanwhile, after comparing the ‘Middle-risk’ and ‘Low-risk’ group, we found that B cells naïve, Plasma cells, T cells CD8, T cells CD4 memory resting enriched in ‘Middle-risk’ group and monocytes, macrophages M0, mast cells resting and neutrophils were enriched in the ‘Low-risk’ group (Fig. 4C). Furthermore, B cell naïve, T cells CD4 memory resting, T cells CD4 memory resting, T cell CD4 memory activated and macrophages M2 had significantly variant enrichment levels between the ‘High-risk’ and the ‘Middle-risk’ groups (Fig. 4D).

To demonstrate the correlation between the ferroptosis and the immune cells infiltration, the relationship between the 24 FRGs expressions and the infiltration degree of 21 variant types of immune cells expect resting dendritic cells undetected in all samples was calculated with the Pearson correlation coefficient (Fig. 4E). The results showed that the expression of SAT1 enabled the ferroptosis enhancement regulated by TP53 was found positive to the infiltration of neutrophils (*R* = 0.816) while negative to the infiltration of naïve B cells (***R*** = -0.584) and plasma cells (***R*** = -0.519).

In order to further describe the heterogeneity of the immune cell infiltration in general among the three groups, we clustered the 80 samples into five subtypes based on the infiltrative levels of the 22 variant types of immune cells in each sample (C1 to C5). Apparently, there was significant difference (*P* < 0.000) between ‘High-risk’ group and ‘Low-risk’ group. The difference between the ‘Middle-risk’ and the ‘Low-risk’ group was also significant (*P* = 0.001). C2 was mainly enriched in the ‘High-risk’ and the ‘Middle-risk’ groups while C3 was enriched in the ‘Low-risk’ group. More information on the heterogeneity of the immune infiltration amongst groups was shown in Fig. 4F.

### Immune characteristics and response to immune checkpoint inhibitors (ICIs) among subgroups

**Fig. 5:**
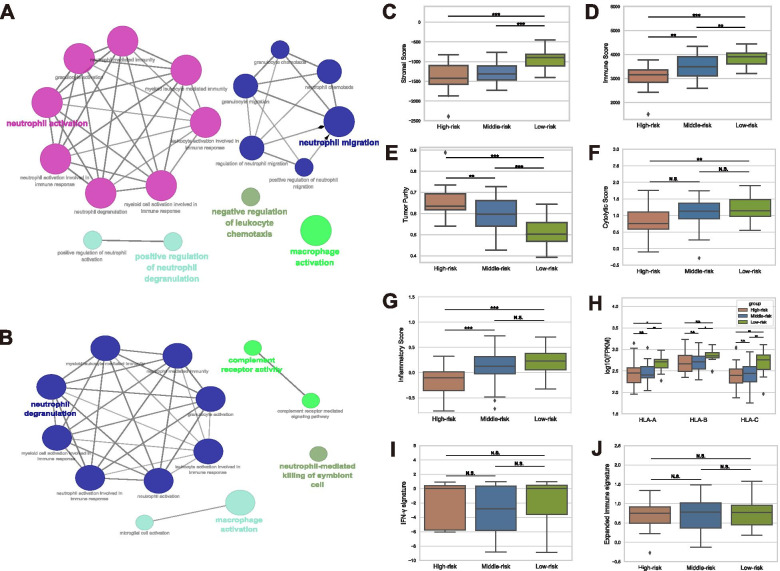
Leukemia’s microenvironment analysis and the response to immune checkpoint inhibitors (ICIs). **A** The immune enrichment analysis of differential expression genes (DEGs) from the comparison result between ‘High-risk’ group and ‘Low-risk’ group. **B** The immune enrichment analysis result of DEGs between ‘Middle-risk’ and ‘Low-risk’ group. **C**-**E** The variances of the leukemia microenvironment were reflexed in stromal score, immune score and tumor purity powered by ‘ESTIMATE’ algorithm. **F**, **G** The specific killing effect of cytotoxic T lymphocytes was evaluated with cytolytic score and inflammatory score. **H** The HLA expression level differences among three groups were shown. **I**, **J** There was no significant difference of the scores of IFN-γ signature or expanded immune signature among three groups which indicated the patients from variant groups had the similar responses to PD-1 blockers

With immune therapy cropping up in cancer therapy, the investigation aiming at tumor micro environment revealed expected values. After analyzing DEGs with ‘ClueGo’ package in Cytoscape software v3.8, the differences between the ‘High-risk’ and the ‘Low-risk’ groups mainly enriched in neutrophil activation, neutrophil migration, positive regulation of neutrophil degranulation, negative regulation of leukocyte chemotaxis and macrophage activation immune signal pathways (Fig. 5A). On the other hand, the comparison between the ‘Middle-risk’ and the ‘Low-risk’ groups, neutrophil degranulation, complement receptor activity, neutrophil-mediated killing of symbiont cell and macrophage activation immune signal pathways were mainly enriched (Fig. 5B).

‘ESTIMATE’ R package was an ideal tool to describe the immune statement of cancers based on gene transcription data. After processing the gene expression data of 80 samples with ‘ESTIMATE’ algorithm, the stromal score, the immune score and the tumor purity were calculated. The ‘Low-risk’ group ranked significantly higher in stromal score, immune score and lower tumor purity compared to the ‘High-risk’ and the ‘Middle-risk’ groups (Fig. 5C-E). These results suggested that the ‘High-risk’ and the ‘Middle-risk’ groups had relatively fewer immune cells in tumor micro-environment (TME) which benefited for the living of leukemia.

Furthermore, the function of T cells attacking against leukemia with cytolytic score and inflammatory score was estimated. There were significant differences in cytolytic scores and inflammatory scores between ‘High-risk’ and ‘Low-risk’ groups (Fig. 5F, G) suggesting the malfunction of T cells in TME as one explanation of the relatively poor prognosis of the ‘High-risk’ groups. Oppositely, the HLA expression was often reduced in cancers for escaping the immune surveillance. In our study, the HLA expression (including HLA-A, B, C) was relatively lower in the ‘High-risk’ and the ‘Middle-risk’ group compared to the ‘Low-risk’ group (Fig. 5H).

Immune checkpoint inhibitors (ICIs) have been recognized as a promoting therapy in solid tumors. However, the role of ICIs in leukemia is still doubtful. Referred to the IFN-γ signature and expanded immune signature, there were no significant difference of the clinical response to PD-1 blockage among three groups (Fig. 5I, J).

### Significant differences in gene enrichment analysis

**Fig. 6:**
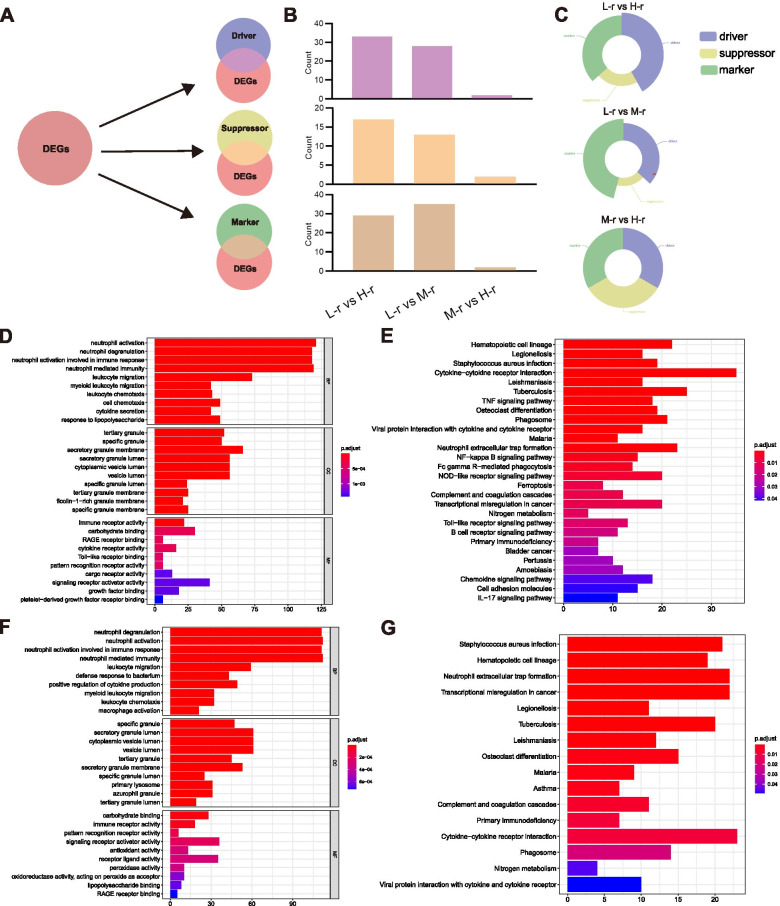
Ferroptosis and GO/KEGG pathway enrichment of DEGs. **A** The different expression genes (DEGs) (|logFC| > 1, FDR < 0.05) resulted from the comparison among three variant risk clusters through the R package edgeR were merged with ferroptosis regulated genes from FerrDb to do the ferroptosis enrichment. **B**, **C** Compared to the DEGs between the ‘Middle-risk’ (M-r) and the ‘Low-risk’ groups (L-r), there were more ferroptosis regulated genes enriching in the DEGs between the ‘High-risk’ (H-r) or ‘Middle-risk’ (M-r) and the ‘Low-risk’ groups. Meanwhile, in three types of ferroptosis regulated genes, ferroptosis driver genes were dominant in the results of L-r vs. H-r while ferroptosis marker genes were the main component after the comparison between L-r and M-r. **D**, **E** The GO and KEGG pathway enrichment of DEGs (|logFC| > 2, FDR < 0.05) between ‘High-risk’ group and ‘Low-risk’ group. **F**, **G** The GO and KEGG pathway enrichment of DEGs (|logFC| > 2, FDR < 0.05) between ‘Middle-risk’ group and ‘Low-risk’ group

In an attempt to explore the mechanism of ferroptosis influencing the prognosis of Ph-neg B-ALL patients, we merged the DEGs between any two groups with the ferroptosis correlated genes from FerrDb (Fig. 6A). As expected, the most notable result was that the ferroptosis correlated genes tended to enrich in the comparison between the ‘High-risk’, the ‘Middle-risk’, and the ‘Low-risk’ groups (Fig. 6B). Furthermore, the ferroptosis driver genes were the dominant genes in enriched ferroptosis correlated genes when compared with ‘High-risk’ and ‘Low-risk’ groups. Meanwhile, as a result of the comparison between the ‘Middle-risk’ and the ‘Low-risk’ groups, ferroptosis marker genes were the most dominant. The equal number of ferroptosis correlated genes in the DEGs between ‘High-risk’ and ‘Middle-risk’ groups (Fig. 6C).

Gene oncology (GO) and KEGG pathway enrichment of DEGs were performed between the ‘High-risk’, ‘Middle-risk’, and ‘Low-risk’ groups. In the enrichment results between ‘High-risk’ and ‘Low-risk’ groups, biological process (BP) term ‘neutrophil activation’, cellular components (CC) term ‘tertiary granule’, and molecular function (MF) ‘immune receptor activity’ were the most trusted GO term (Fig. 6D). Meanwhile, ‘Hematopoietic cell lineage’ was the most trusted KEGG pathway term (Fig. 6E). correspondingly, the biological process (BP) ‘neutrophil degranulation’, cellular components (CC) ‘specific granule’, molecular function (MF) ‘carbohydrate binding’ and KEGG pathway ‘*Staphylococcus aureus* infection’ terms were the most significantly enriched terms in the results when comparing the ‘Middle-risk’ and the ‘Low-risk’ groups (Fig. 6F, G).

### Identification of the potential implementation of Sorafenib in ‘high-risk’ Ph-neg B-ALL

Since Sorafenib is proved as a drug that is able to induce ferroptosis in cancers and also being implemented in the treatment of AML, we further explored the potential implement of Sorafenib in ALL.

**Fig. 7:**
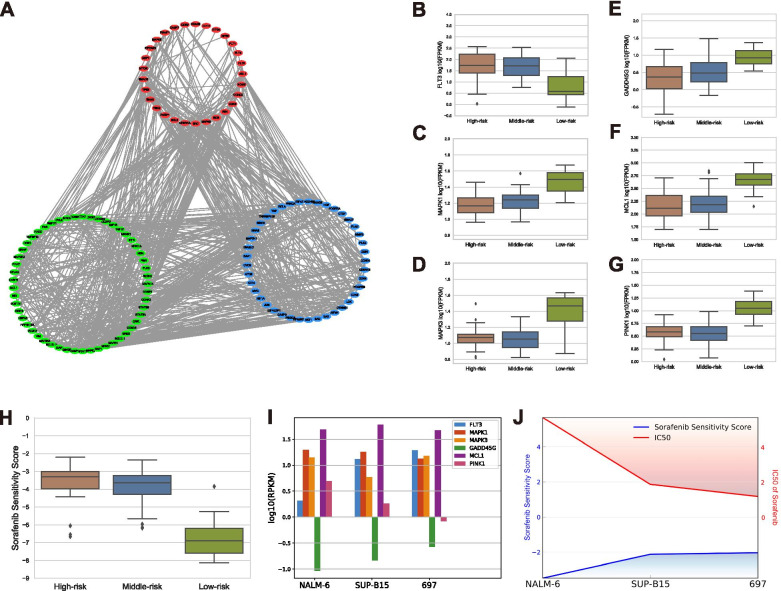
Sorafenib was identified as a ferroptosis inducer expected to treat high-risk Ph-neg B-ALL patients. **A** The protein-protein interaction network composed of the genes correlated Sorafenib. (Red: significantly up-regulated genes in ‘High-risk’ group or ‘Middle-risk’ group; green: significantly down-regulated genes in ‘High-risk’ group or ‘Middle-risk’ group; blue: the genes with no significance among three groups) **B**-**G** The expression of six genes correlated with the susceptibility of Sorafenib among three clusters. **H** The Sorafenib sensitivity scores of the samples in three groups calculated with the expression of six Sorafenib sensitivity related genes. **I** Sorafenib sensitivity related gene expression of three representative B-ALL cell lines recorded in CCLE database were shown in the form of log10(RPKM). **J** Combined the gene expression of the three cell lines, each cell line was ranked with a sorafenib sensitivity score (blue). Analyzed with the sensitivity of three cell lines to Sorafenib (red, evaluated with IC50), we validated the higher the sorafenib sensitivity score was, the more sensitive to Sorafenib B-ALL was

Firstly, we compared the Sorafenib related genes acquired from the CTD database between ‘High-risk’ and ‘Low-risk’ groups. The comparison of the Sorafenib related genes between ‘Middle-risk’ and ‘Low-risk’ groups was done in the same way. After the comparisons, the genes which expressed highly in both the ‘High-risk’ and the ‘Middle-risk’ groups were recognized as the up-regulated genes. Meanwhile, the genes expressed highly in the ‘Low-risk’ group in the both comparisons were defined as the down-regulated genes. The rest Sorafenib related genes were classified as the not-significant genes. As a result, we totally acquired 31 up-regulated genes (red, ***P*** < 0.05), 56 down-regulated genes (green, ***P*** < 0.05) and 45 not-significant genes (blue, ***P*** ≥ 0.05). High-confident interaction score (> 0.9) Protein-Protein Interaction analysis (PPI) of these Sorafenib correlated genes were performed referred to STRING database (http://string-db.org/) (Fig. 7A).

Afterwards, we selected six genes involved in the susceptibility of Sorafenib. They were FLT3 positively correlated to the sensitivity of Sorafenib and MAPK1, MAPK3, GADD45G, MCL1, PINK1 and negatively related to the sensitivity of Sorafenib. Their expression differences between three groups were shown in Fig. 7B-G. In order to speculate the sensitivity of Sorafenib by combining these factors, we calculated the Sorafenib sensitivity score using the equation: log10 (FLT3)-log10 (MAPK1)-log10 (MAPK3)-log10 (GADD45G)-log10 (MCL1)-log10 (PINK1). Intriguingly, compared to ‘Low-risk’ group, ‘High-risk’ and ‘Middle-risk’ groups were ranked as higher scores which indicated Ph-neg B-ALL patients in ‘High-risk’ or ‘Middle-risk’ groups may be susceptible to the treatment with Sorafenib (Fig. 7H).

In the aim of verifying the efficiency of our established Sorafenib sensitivity score, we downloaded six gene expression data of the ALL cell lines NALM-6, SUP-B15 and 697 from CCLE database (Fig. 7I) and then calculated the Sorafenib sensitivity score of the three lines. Correlated the real sensitivity in IC50 of these cell lines on GDSC database, we surprisingly found that with the increasing of Sorafenib Sensitivity Score, the IC50 of these cell lines decreased. These data proved that our score system was an effective tool to assess the susceptibility of Sorafenib and Sorafenib may help to reverse the poor prognosis of Ph-neg B-ALL patients in ‘High-risk’ and ‘Middle-risk’ groups.

### FRGs-based model in help of survival evaluation of Ph-neg B-ALL

**Fig. 8:**
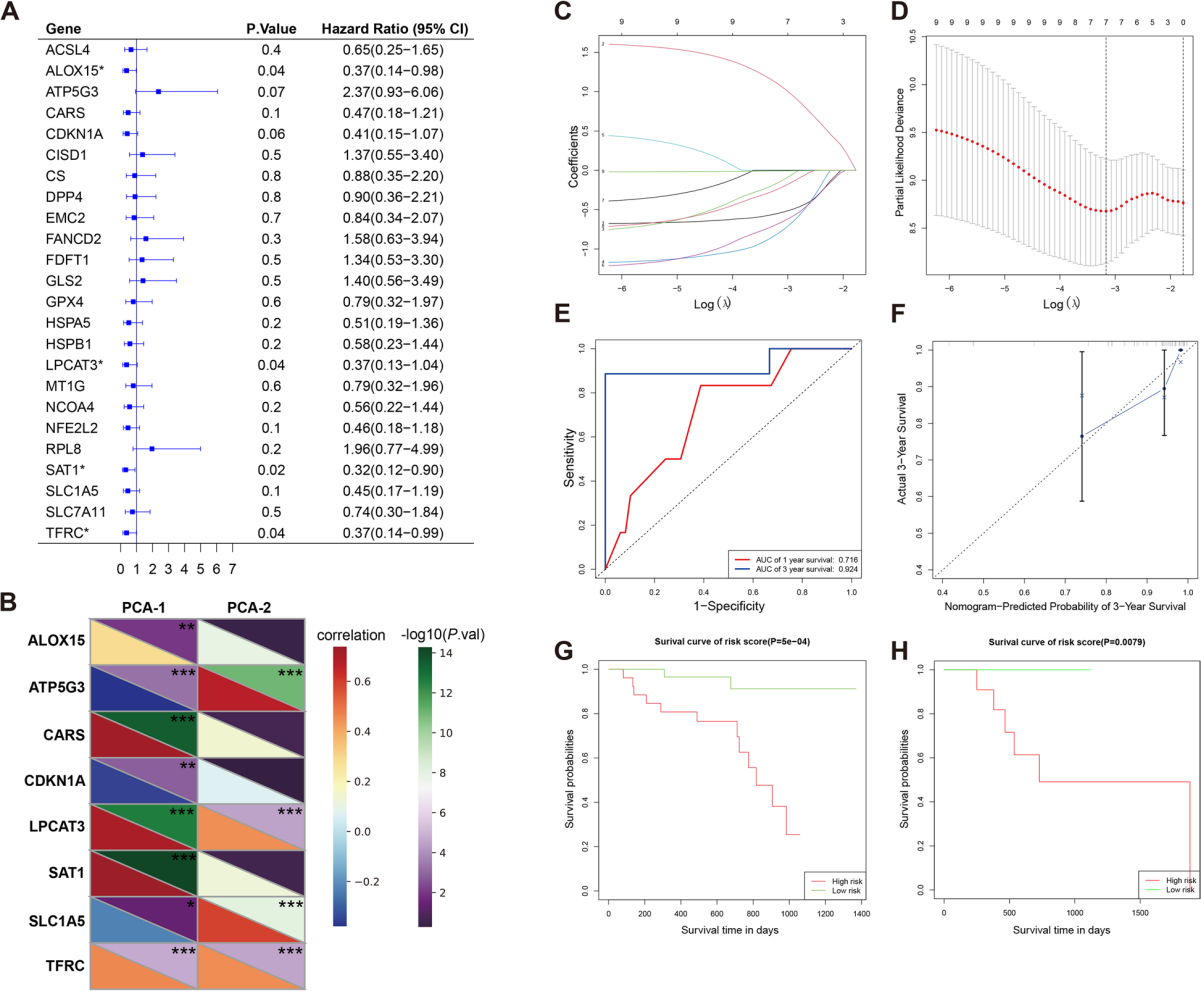
The development and validation of survival predicted model based on 8 FRGs. **A** Univariate Cox regression analysis of 24 FRGs. **B** Correlation betweenPCA1/2 and 8-survival correlated FRGs. **C** LASSO regression of the 8 survival-correlated FRGs. **D** Cross-validations for tuning the parameter selection in the LASSO regression. **E**, **F** Both ROC curves and the calibration demonstrated the predictive efficiency of the model. **G**, **H** Kaplan–Meier curves for the overall survival of variant risk patients from the development cohort and the validation cohort

As our previous analysis had demonstrated that the 24 FRGs enabled to cluster the Ph-neg B-ALL samples into variant-risk groups, univariate Cox regression was further employed to screen the FRGs influencing survival (Fig. 8A). The 8 genes (ALOX15, ATP5G3, CARS, CDKN1A, LPCAT3, SAT1, SLC1A5 and TFRC) that met the criteria of ***P*** < 0.1 were retained for modeling. The correlation between the 8 genes and the parameters of the former K-means clustering were displayed in the form of heat map (Fig. 8B). Through employing the LASSO Cox regression analysis, an 8-gene signature was constructed referred to the optimum λ value (Fig. 8C, D). Time-dependent receiver operating characteristic (ROC) was adopted as a classical method to evaluate the sensitivity and the specificity of the prognostic models. After calculating, the AUC was 0.716 for 1 year and 0.924 for 3 year in the development cohort (Fig. 8E). On the other hand, the calibration curve was utilized to evaluate a 3-year prediction efficiency of our established 8-gene multivariate Cox regression model (Fig. 8F). In the end, our model enabled to distinguish samples of favorable and poor prognosis in both development and validation groups after Kaplan-Meier analysis (Fig. 8G, H).

## Discussion

In this study, we retrospectively collected clinical data of 80 Ph-neg B-ALLs treated in our center and the results revealed that the combination of 24 FRGs expressions enabled to cluster these patients into three groups using the unsupervised machine learning algorithm Kmeans clustering. Moreover, there was a significant correlation between the immune microenvironment and FRG-based clustering. In addition, the variant frequency of the ferroptosis regulated gene mutations among these groups and the gene functional enrichment mechanism were explored to explain these differences. Based on our findings on the ferroptosis related heterogeneity among the groups, we speculated and validated that Sorafenib might be an effective drug to improve the poor prognosis of high-risk Ph-neg B-ALLs.

Ferroptosis is a recently discovered programming death in the characteristics of cell membrane damage due to the GPX4 loss of activity and the intracellular accumulation of lipid reactive oxygen [25]. Accumulated evidence showed that the ferroptosis widely participates in tumorigenesis and plays a promoting role in tumor therapy [26, 27]. However, there is a lack of studies highlighting ferroptosis in B cell acute lymphoblastic leukemia. Moreover, the outcome of Ph-neg B-ALL after treatment was broadly variant due to lack of targeted therapy like imatinib in Philadelphia positive ALL. Here, we employed NGS technology to explore the heterogeneity in both genetic and transcriptional levels of Ph-neg B-ALLs with 24 FRGs. Not only did we identify the expression of FRGs in ALL were widely different from the normal people, but we also established that the combination of FRGs expression had the ability to recognize variant risk groups of Ph-neg B-ALL powered by Kmeans clustering. These results were the clinical reflection of evidences that the progression of leukemia is highly reliant on iron which maintains the rapid growth rate of leukemia cells [28–30].

Emerging immune check checkpoint inhibitors (ICIs) based therapy raised our attention to investigate the leukemia’s immune microenvironment. However, the manner which the ferroptosis mechanism involves in leukemia immune microenvironment still in need for more research, our results have indicated the microenvironment aberrance accompanied by ferroptosis difference. Compared to the ‘Middle-risk’ and the ‘Low-risk’ groups; there was more B cells naïve infiltration related with higher burden of leukemia in ‘High-risk’ groups. Moreover, CD4+ memory activated T cells significantly decreased in the ‘High-risk’ groups; it may attenuate the ferroptosis of leukemia induced by cytotoxic T cells according to previous reports [31]. In addition, the polarization of macrophages to an M2 phenotype was significantly outstanding in the ‘High-risk’ group. This phenomenon may involve in ferroptosis and leaded to stimulate leukemia growth ultimately [32]. Furthermore, since there was little known about the role of ICIs in ALL, our research indicated that there was no potentially higher benefits from PD-1 blockage therapy in the ‘High-risk’ or ‘Middle-risk’ groups compared to the ‘Low-risk’ group. These results raised the idea of the implementation of ferroptosis inducers in the ‘High-risk’ and the ‘Middle-risk’ groups which involved low immune cell infiltration. For one thing, leukemia microenvironment with low immune cell infiltration facilitates the leukemia to escape the surveillance of the immune system [33]. On the other hand, the inflammatory environment induced by ferroptosis inducers plays the chemotaxis effect to immune cells and changes the ‘cold’ leukemia to the ‘hot’ leukemia which is vulnerable to the chemo-therapy.

One of the reasons why ferroptosis closely participated in Ph-neg B-ALL is it has relatively high frequency of RAS and TP53 gene mutations which was involved in ferroptosis [34–36]. Based on these findings, the mutation of ferroptosis driver genes were significantly concentrated in the ‘High-risk’ group which may induce the ferroptosis resistance to leukemia. To demonstrate our hypothesis, we chose Sorafenib as the promoting curable drug for its ferroptosis inducing effect [37] and successful implementation in FLT3-ITD positive AML [38]. In past studies, Sorafenib was found to induce ferroptosis mainly by inhibiting the activity of system xc- and not necessarily on the inhibition of its kinase targets [37, 39, 40]. However, there were few reported clinical trials of Sorafenib in Ph-neg B-ALL patients. In our investigation, it was surprising to point that patients from the ‘High-risk’ and the ‘Middle-risk’ groups based on gene expression were predicted to be more sensitive to Sorafenib possibly owing to variant degrees of ferroptosis involvement in Ph-neg B-ALL. Based on these interesting findings, we will further to initiate a clinical trial to verify the curable effect of Sorafenib combined with chemotherapy in high-risk Ph-neg B-ALLs.

Comparable to other models correlated with ferroptosis in other types of cancers [11, 41], we systematically investigated the ferroptosis involvement in our samples and proved that the ferroptosis plays an important but still an undiscovered role in Ph-neg B-ALL. Although the prognosis of Philadelphia positive B-ALL patients became favorable thanks to the invention of imatinib, a considerable number of Philadelphia negative B-ALL patients showed less sensitivity to the common therapy especially in adult patients. Moreover, lack of prognosis evaluation system impeded the clinical Individualized treatment. Herein, based on 8 FRGs after sorting, we created an efficient Cox regression model to estimate the prognosis of Ph-neg B-ALL patients.

According to these findings and based on the data collected from the patients treated in our hospital and checked by at least two persons separately, our results deducted from the DNA variance and RNA level quantifications for FRGs should be validated in a larger and perspective cohort in the future. Moreover, ferroptosis is a newly discovered and complex biological process which needs to be further investigated. In addition, due to the rapid improvement in B-ALL treatment, especially after the coming of CAR-T therapy, we faced limited cases to analyze the importance of ferroptosis in Ph-neg B-ALL patients and the exploration of ferroptosis seemed to be meaningful in the new age since some patients enrolled in our study had received CAR-T therapy. Consequently, our study based on the current knowledge and data mining might need further updates and more validations in researches to come.

## Conclusion

In summary, after the exploration the potential role of ferroptosis in Ph-neg B-ALL with the clinical data and the RNA-seq results of 80 Ph-neg B-ALL treated in our center. Not only did we demonstrated the combination of expression of FRGs enabled to cluster Ph-neg B-ALL patients into variant risk groups, but also analyzed their correlation with leukemia’s immune microenvironment and gene mutation characteristics. Based on these findings, we further raised the potential implementation of Sorafenib in high-risk Ph-neg ALL patients. Moreover, a Cox regression model based on 8 FRGs was established to help evaluate the prognosis of Ph-neg B-ALL patients. Overall, our research put forward a new view to understand the pathogenesis of Ph-neg ALL and evaluate the involved patients.

## Supplementary Information

**Additional file 1: Table S1.** Baseline clinical characteristics of Ph-neg B-ALL patients according to K-means clustering.

**Additional file 2: Table S2.** Genomic mutation detection panel.

**Additional file 3.**

## Data Availability

The gene expression data in the form of FPKM values analyzed during the current study are attached in the additional supporting file (matrix_for_analysis.txt).

## Abbreviations

Ph-neg B-ALL: Philadelphia chromosome-negative B cell acute lymphoblastic leukemia
FRG: Ferroptosis related gene
TKI: Tyrosine kinase inhibitor
B-ALL: B cell acute lymphoblastic leukemia
CAR-T: Chimeric antigen receptor T cells
R/R ALL: Refractory or relapse acute lymphoblastic leukemia
ROS: Reactive oxygen species
FISH: Fluorescence in situ hybridization
RNA-seq: Whole transcription sequencing
qPCR: Fluorescent quantitative polymerase chain reaction
FPKM: Fragments per kilobase million mapped reads
CDS: Coding sequence
PCA: Principal component analysis
ARI: Adjusted rand index
AMI: Adjusted mutual index
FMI: Fowlkes–Mallows index
DEG: Different expression gene
CTD: Comparative Toxicogenomics Database
CCLE: Broad Institute Cancer Cell Line Encyclopedia
IC50: Half maximal inhibitory concentration
GDSC: Genomics of Drug Sensitivity in Cancer
HR: Hazard ratio
AUC: Area under curve
LASSO: Least absolute shrinkage and selection operator
CBC: Complete blood count
HSCT: Hematopoietic stem cell transplantation
NGS: Next generation sequencing
GO: Gene oncology
PPI: Protein-protein interaction analysis
ROC: Receiver operating characteristic
