# Supplementary material for: Identification of immune subtypes of Ph-neg B-ALL with ferroptosis related genes and the potential implementation of Sorafenib
Source: BMC Cancer. 2021 Dec 14;21:1331. doi: 10.1186/s12885-021-09076-w (PMC8670244; doi:10.1186/s12885-021-09076-w)
Supplement: Supplementary file 1 — Additional file 1: Table S1. Baseline clinical characteristics of Ph-neg B-ALL patients according to K-means clustering. [file 12885_2021_9076_MOESM1_ESM.docx]

**Table S1.** Baseline clinical characteristics of Ph-neg B-ALL patients according to K-means clustering

|  |  | **High-risk (n=25)** | | | | **Middle-risk (n=39)** | | | | | **Low-risk (n=16)** | | | | ***P*** |
| --- | --- | --- | --- | --- | --- | --- | --- | --- | --- | --- | --- | --- | --- | --- | --- |
| **Sex** | |  |  | | |  | |  | | |  | |  | | .7517 |
|  | Female | 11 | | (44%) | | | 21 | | (54%) | | | 8 | | (50%) |  |
|  | Male | 14 | | (56%) | | | 18 | | (46%) | | | 8 | | (50%) |  |
| **Age at diagnosis** (years) | | 20 | | (9-56) | | | 29 | | (10-54) | | | 26 | | (6-48) | .7950 |
| **CBC** | |  |  | | |  | |  | | |  | |  | |  |
|  | WBC (10^9^/L) | 11.7 | | (1.2-100) | | | 7.0 | | (1.05-102) | | | 6.1 | | (0.9-56) | .6967 |
|  | Hb (g/L) | 74.0 | | (37-164) | | | 76.0 | | (42-129) | | | 82.0 | | (37-118) | .6281 |
|  | Plt (10^9^/L) | 52.0 | | (17-257) | | | 61.0 | | (2-329) | | | 70.0 | | (4-341) | .7402 |
| **Blasts** (%) | | 84.0 | | (53-95) | | | 84.8 | | (50-99) | | | 81.0 | | (62-96) | .8747 |
| **Immunology** (%) | |  |  | | |  | |  | | |  | |  | | .4157 |
|  | Pre-B ALL | 2 | | (8%) | | | 4 | | (10%) | | | 2 | | (13%) |  |
|  | Pro-B ALL | 6 | | (24%) | | | 14 | | (36%) | | | 3 | | (19%) |  |
|  | Immature B ALL | 0 | | (0%) | | | 4 | | (10%) | | | 1 | | (6%) |  |
|  | Unclassified | 17 | | (68%) | | | 17 | | (44%) | | | 10 | | (62%) |  |
| **Cytogenetics** | |  |  | | |  | |  | | |  | |  | | >.9999 |
|  | Normal | 13 | | | (52%) | 25 | | | | (64%) | 7 | | | (44%) |  |
|  | Others | 5 | | | (20%) | 6 | | | | (15%) | 4 | | | (25%) |  |
|  | complex karyotype | 4 | | | (16%) | 3 | | | | (8%) | 1 | | | (6%) |  |
|  | insufficient metaphases | 3 | | | (12%) | 5 | | | | (13%) | 4 | | | (25%) |  |
| **Molecular Alterations** | |  |  | | |  | |  | | |  | |  | | **.0269^*^** |
|  | CDKN2A or CDKN2B deletion | 1 | | | (4%) | 1 | | | | (2%) | 0 | | | (0%) |  |
|  | PAX5 alteration | 3 | | | (12%) | 2 | | | | (5%) | 1 | | | (6%) |  |
|  | TP53 mutation | 2 | | | (8%) | 4 | | | | (10%) | 2 | | | (13%) |  |
|  | JAK2 mutation | 0 | | | (0%) | 2 | | | | (5%) | 0 | | | (0%) |  |
|  | NRAS mutation | 7 | | | (28%) | 9 | | | | (23%) | 2 | | | (13%) |  |
|  | KRAS mutation | 4 | | | (16%) | 6 | | | | (15%) | 1 | | | (6%) |  |
|  | SETD2 mutation | 5 | | | (2%) | 4 | | | | (10%) | 2 | | | (13%) |  |
|  | PTPN11 mutation | 3 | | | (12%) | 3 | | | | (8%) | 0 | | | (0%) |  |
| **CAR-T therapy** | |  |  | | |  | |  | | |  | |  | | >.9999 |
|  | Yes | 5 | | | (20%) | 12 | | | | (31%) | 7 | | | (44%) |  |
|  | No | 20 | | | (80%) | 27 | | | | (69%) | 9 | | | (56%) |  |
| **HSCT** | |  |  | | |  | |  | | |  | |  | | >.9999 |
|  | Yes | 13 | | | (52%) | 31 | | | | (79%) | 12 | | | (75%) |  |
|  | No | 12 | | | (48%) | 8 | | | | (21%) | 4 | | | (25%) |  |

**WBC**: white blood cells; **Hb**: hemoglobin; **Plt**: platelet; **CAR-T**: chimeric antigen receptor T cells; **HSCT**: hematopoietic stem cell transplantation.
